# Origins and Neurochemical Complexity of Preganglionic Neurons Supplying the Superior Cervical Ganglion in the Domestic Pig

**DOI:** 10.1007/s12031-014-0321-8

**Published:** 2014-05-23

**Authors:** Judyta K. Juranek, Joanna A. Wojtkiewicz

**Affiliations:** 1 Department of Medicine, New York University Medical Center, 550 First Avenue, Smilow 906, New York, NY USA; 2 Department of Pathophysiology, University of Warmia and Mazury, Olsztyn, Poland; 3 Department of Neurology and Neurosurgery, Division of Neurosurgery, University of Warmia and Mazury, Ul. Warszawska 30, 10-082 Olsztyn, Poland; 4 Stem Cell Research Laboratory, Faculty of Medical Sciences, University of Warmia and Mazury, Olsztyn, Poland

**Keywords:** Sympathetic preganglionic neurons, Superior cervical ganglion, Domestic pig, Retrograde tracing, Neurochemical coding

## Abstract

The superior cervical ganglion (SCG) is a center of sympathetic innervation of all head and neck organs. SCG sympathetic preganglionic neurons (SPN) were found in the nucleus intermediolateralis pars principalis (IMLpp), the nucleus intermediolateralis pars funicularis (IMLpf), the nucleus intercalatus spinalis (IC), and the nucleus intercalatus spinalis pars paraependymalis (ICpe). Despite its importance, little is known of SCG innervation and chemical coding in the laboratory pig, a model that is physiologically and anatomically representative of humans. Here in our study, we established the distribution and chemical coding of Fast Blue (FB) retrogradely labelled SPN innervating porcine SCG. After unilateral injection of FB retrograde tracer into the left SCG, labeled neurons were found solely on the ipsilateral side with approximately 98 % located in Th_1_–Th_3_ segments and predominantly distributed in the IMLpp and IMLpf. Neurochemical analysis revealed that approximately 80 % of SPN were positive both to choline acetyltransferase (ChAT) and nitric oxide synthase (NOS) and were surrounded by a plethora of opioidergic and peptiergic nerve terminals. The results of our study provide a detailed description of the porcine preganglionic neuroarchitecture of neurons controlling the SCG, setting the stage for further studies concerning SPN plasticity under experimental/pathological conditions.

## Introduction

The superior cervical ganglion (SCG) is the center of sympathetic innervation of all head and neck organs. It provides sympathetic input to numerous key organs and structures such as the salivary glands, pineal gland, thyroid, carotid body, choroid plexus, brain and cranial muscle vasculature, eyes, and lacrimal gland, thus playing a vital role in a number of neuroendocrine, visceral, or vision-related processes (Cardinali et al. 1983). Studies showed that SCG is involved in circadian rhythm regulation (Lingappa and Zigmond 2013), thyroid function modulation (Young et al. 2005), tear production (Ding et al. 2003; Tangkrisanavinont 1984), skin vasoconstriction (Gibbins et al. 1998), and cerebral arterial supply regulation (Arbab et al. 1986; Cassaglia et al. 2008; Goadsby 2013). Despite being considered a small neuroendocrine center (Cardinali et al. 1983), a complex neurochemical phenotype of its preganglionic neurons and fibers has yet to be completed.

In animal models studied so far, sympathetic preganglionic neurons (SPN) supplying the SCG were found in cervico-thoracic neuromeres, which were confined to the following nuclei: (1) nucleus intermediolateralis pars principalis (IMLpp), (2) nucleus intermediolateralis pars funicularis (IMLpf), (3) nucleus intercalatus spinalis (IC), and (4) nucleus intercalatus spinalis pars paraependymalis (ICpe) (Dalsgaard and Elfvin 1979; Petras and Faden 1978; Yau et al. 1991); however, until now there is no data clarifying the origins of porcine SCG innervation.

Studies conducted in laboratory animals show that the chemical phenotype of these neurons is very diverse and complex, underscoring the importance of the ganglion in autonomic regulation. Acetylcholine (ACh) is the main SPN neurotransmitter; however, studies show that its actions upon SCG activity are largely supported by nitric oxide (NO) present in preganglionic neurons alongside ACh (Okamura et al. 1995).

It has been shown that NO modulates cyclic GMP synthesis in cholinergic neurons, affecting ACh release from these neurons and thus regulating overall ACh activity (Prast et al. 1995).

Furthermore, data obtained from human and small animal laboratory species reveal that aside from ACh and NO, a number of other neuromodulatory substances such as serotonin (Jensen et al. 1995), leu-enkephalin (Klimaschewski et al. 1995), pituitary adenylate cyclase-activating polypeptide (PACAP) (Beaudet et al. 1998), neuropeptide (NPY), calcitonin gene-related peptide (CGRP) (Yamamoto et al. 1989), substance P (SP) (Klimaschewski et al. 1995; Llewellyn-Smith et al. 1997; Tan et al. 1996), and vasoactive intestinal peptide (VIP) (Sasek et al. 1991) are present in the SCG-innervating preganglionic neurons, likely contributing to the SCG neuroendocrine function.

Here in the present study, we investigated the distribution and neurochemical coding of SCG supplying neurons in the pig, an animal model physiologically and anatomically similar to humans. In our previous study, we showed a complete 2D reconstruction of porcine SCG and provided neurochemical characteristics of SCG neurons supplying the porcine parotid gland (Wojtkiewicz et al. 2011). In this project, we have advanced in our research, paving the way to the source of SCG innervation and providing a detailed overview of SCG preganglionic neuron distribution and their complex neurochemical phenotype.

## Materials and Methods

Six 8-week-old female pigs of the Large White Polish breed, weighing ca. 20–25 kg, were used in the study. The animals were housed in accordance with the principles of Laboratory Animal Care (NIH publication no. 86-23, revised 1985) and approved by the Ethical Commission of Veterinary Medicine Faculty at the University of Warmia and Mazury, Olsztyn, Poland (Resolution No. 47/2006). The animals were preanesthetized with propionyl promazine (0.4 mg/kg b/w, i.m.) and deeply anesthetized with sodium barbital (25 mg/kg b/w; i.v.). The left SCG, exposed via midline neck incision, was injected with retrograde tracer, Fast Blue (FB, EMS, Grivory, Deutschland, GMbH, Postfach) at multiple sites along the SCG. Injections were given with a Hamilton microsyringe at a total volume of 20 μl 5 % dye solution (1 μl dye solution per injection). After a 3-week survival time, animals were re-anesthetized and sacrificed by an overdose of pentobarbital. Following sacrifice, animals were perfused transcardially with heparinized physiological saline in 0.1 phosphate buffer (PB, pH 7.4) and fixative solution containing 4 % paraformaldehyde 0.1 M PB (pH 7.4). Immediately after perfusion, spinal cord C5–Th8 segments were exposed by laminectomy and dissected. The segments were immersed in the same fixative for 20 min and stored in 18 % phosphate-buffered sucrose solution for further processing. The spinal cord segments were sectioned transversally (*n* = 3) and longitudinally (*n* = 3) on the cryostat at 10 μm in thickness, mounted on glass slides, and stored at 70 °C.

Immunofluorescent staining was performed according to standard procedure as described (Wojtkiewicz et al. 2011). Briefly, 10-μm-thick cryostat sections were air-dried at RT for 45 min and rinsed (3 × 15 min) with phosphate-buffered saline (PBS, pH 7.4). Afterwards, the samples were incubated with a blocking mixture containing 1 % Triton X-100 (Sigma-Aldrich, USA), 0.1 % bovine serum albumin (Sigma-Aldrich, USA), 0.05 % thimerosal (Sigma-Aldrich, USA), 0.01 % NaN_3_, and 10 % of normal goat serum (MP Biomedicals, USA) in 0.01 M phosphate-buffered saline for 1 h at RT, rinsed in PBS (3 × 15 min), and incubated overnight with the following primary antibodies: mouse anti-nitric oxide synthase (NOS), co-stained with an array of rabbit antisera against dynorphin A (DYN A), Leu-5 enkephalin (LENK), neuropeptide Y (NPY), α-neoendorphin (αNEO), calbindin D-28 k (CB-D28k), calretinin (CRT), galanin (GAL), vasoactive intestinal peptide (VIP), peptide histidine-isoleucine (PHI-27), vesicular acetylcholine transporter (VAChT), substance P (SP), calcitonin gene-related peptide (CGRP), pituitary adenylate cyclase-activating peptide-27 (PACAP-27), somatostatin (SOM), choline acetyltransferase (ChAT), serotonin (5-HT), cholecystokinin (CCK), cocaine- and amphetamine-regulated transcript (CART), and GABA (for dilutions and secondary antibodies, see Table 1). Sections were then incubated with a mixture of appropriate fluorescein isothiocyanate (FITC)-conjugated secondary antisera and biotinylated goat anti-rabbit antibodies (1 h). The latter antibodies were visualized by additional incubation of sections with streptavidin–CY3 complex (1 h). After staining, the sections were mounted with carbonate-buffered glycerol (pH 8.6) and cover slipped. The specificity of primary antisera was tested as follows: sections were incubated with antibody, preabsorbed with synthetic antigen (10 μg of antigen/ml diluted antiserum); the primary antibody was omitted from the incubation; or normal rabbit or mouse serum was substituted for the primary antibody.

**Table 1:** Detailed list of primary and secondary antibodies used in the study

Antisera	Reagent	Code	Host Species	Dilution	Supplier
Primary antibodies					
ChAT		AB5052	Rabbit	1:10,000	Chemicon Int. Inc, UK; www.chemicon.com
NPY		NA 1233	Rabbit	1:10,000	Biomol Res. Lab. Inc, US; www.biomol.com
SOM		8330-0154	Rabbit	1:10,000	Biogenesis Inc, www.biogenesis.co.uk
VIP		11428	Rabbit	1:10,000	MP Biomedicals; www.mpbio.com
GAL		RIN7153	Rabbit	1:10,000	Bachem AG; www.bachem.com
CB-D28k		CB-38	Rabbit	1:10,000	SWANT, S; www.swant.com
CRT		7699/4	Rabbit	1:20,000	SWANT, S; www.swant.com
NOS		N2280	Mouse	1:2,000	Sigma; http://www.sigmaaldrich.com
LENK		EA 1149	Rabbit	1:10,000	BioReagents Inc, UK; www.bioreagents.com
DYNA		S-4019	Rabbit	1: 10,000	Bachem AG; www.bachem.com
αNEO		S-3149	Rabbit	1:10,000	Bachem AG; www.bachem.com
VAChT		H-V006	Rabbit	1:10,000	Phoenix,; www.phoenixpeptide.com
PACAP		IHC 8922	Rabbit	1:20,000	Bachem AG; www.bachem.com
PHI		S-3130	Rabbit	1:10,000	Bachem AG; www.bachem.com
CGRP		11189	Rabbit	1:10,000	MP Biomedicals; www.mpbio.com
SP		8450-0505	Rabbit	1:10,000	Biogenesis Inc, www.biogenesis.co.uk
5HT		S5545	Rabbit	1:5,000	Sigma, US; www.sigma-aldrich.com
Secondary antibodies					
	Donkey anti-mouse IgG (H + L) conjugated with FITC			1:800	715-095-151; Jackson IR Lab, US; www.jacksonimmuno.com
	CY3-conjugated F(ab’)_2_ donkey anti-rabbit IgG (H + L) -			1:9,000	711-166-152, Jackson IR Lab, US; www.jacksonimmuno.com

Sections were examined under an Olympus BX51 fluorescence microscope equipped with a barrier filter for FB. Microphotographs were acquired with a CCD camera connected to a PC equipped with AnalySIS image analysis software (ver. 3.2; Soft Imaging System GmbH, Münster, Germany). To determine the relative number of FB-positive cells, neurons were counted in every 16th section at × 20 objective. Only neurons with a clearly visible nucleus were considered. Data was pooled from animals and analyzed with GraphPad Prism 5 software (GraphPad Software, La Jolla, CA, USA). Results are presented as means ± standard error of mean (SEM). Nerve fiber distribution was assessed under × 40 objective by subjective observation (two independent researchers) as previously described (Gonkowski et al. 2013) and depending on the density of labeling, described as not found or very few, small, moderate, and large number of nerve fibers.

## Results

Following FB injections of left SCG, FB-positive neurons were found on the ipsilateral side of the spinal cord. A total of 13,013 neurons, combined from longitudinal and transverse sections, were counted. The sympathetic preganglionic neurons (SPN) projecting to the porcine SCG were found in C8 to Th6 neuromeres, with the vast majority of them located in Th1–Th3 segments (94 ± 4.4 %), ranging from 27.6 ± 3.0 % (Th1) to 34.5 ± 4.0 % (Th2) and 31.6 ± 6.3 % (Th3), respectively.

**Fig. 1:**
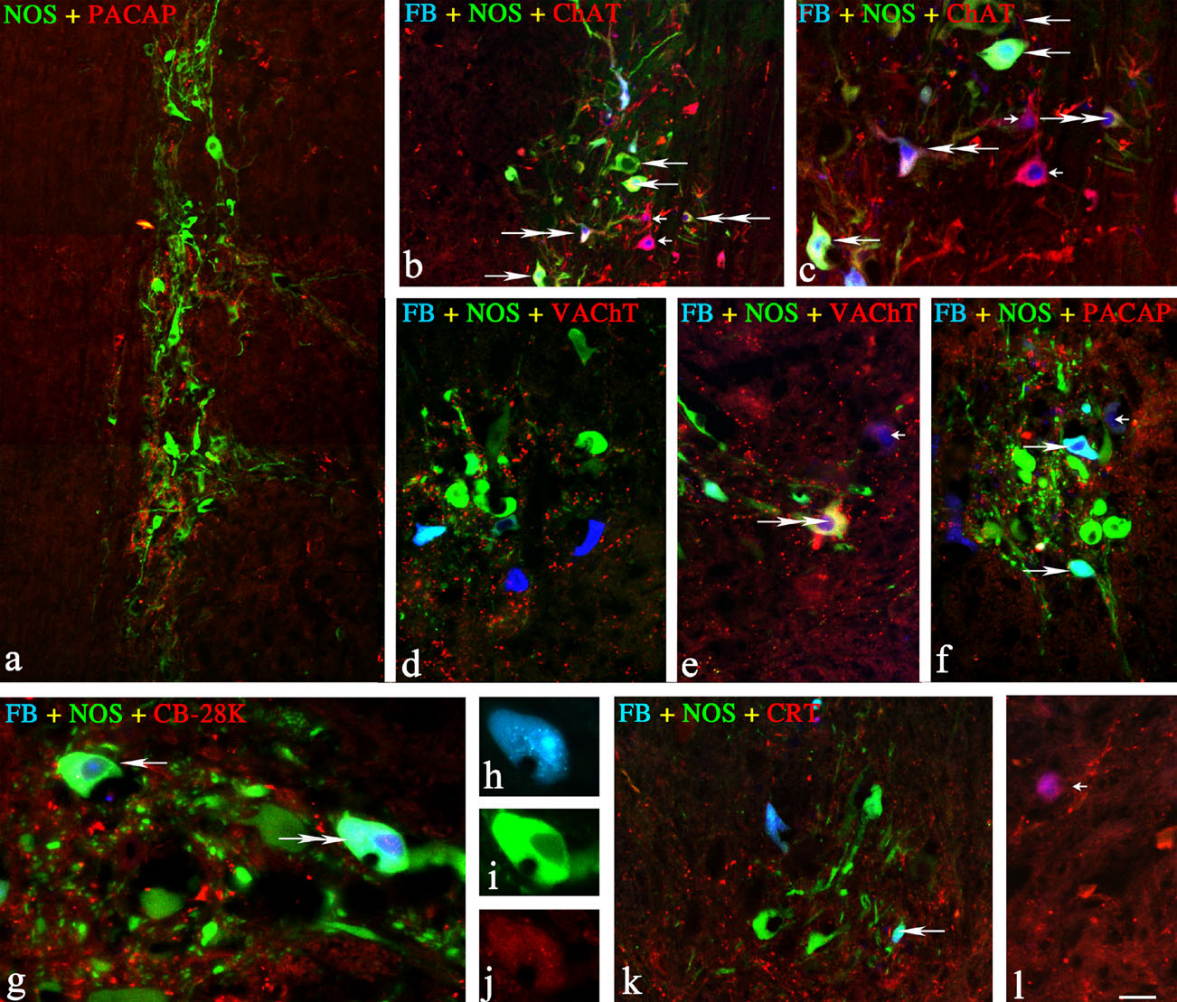
Representative images of SCG-projecting neurons located in the spinal cord IML column. All pictures are composites of merged images taken separately from the red, green, and/or blue fluorescent channels. **a** Double-labelled NOS+/PACAP + neurons in the Th3 neuromere, a compilation of three photos. **b**, **c** Three different populations of NOS/ChAT-positive neurons in Th3 neuromere; FB^+^/NOS^+^ neurons positive for ChAT (*two double arrows*), FB+/NOS + negative for ChAT (*three long arrow*s), and double-labeled FB^+^/ChAT^+^ negative for NOS (*two small arrows*). **d**, **e** Single FB + neurons scattered between a small population of FB^+^/NOS^+^/VAChT + (triple staining, *two double arrows*) and FB+/VAChT + neurons (*small arrow*). **f** A population of double FB^+^/NOS^+^/PACAP^−^ (*two single arrows*) and FB^+^/NOS^−^/PACAP + (*small arrow*) neurons observed in Th3 neuromere. **g**, **j** The FB^+^/NOS^+^/CB-28 k^+^ (*one double arrow*) and FB^+^/NOS^+^/CB-28 k^−^ (*single arrow*) neurons observed in Th3 neuromere (merged image). Enlarged images of the triple-stained neuron, split channels: **h** blue—FB labeling. **i** Green—NOS labeling. **j** Red—CB-28 K labeling. **k** The FB^+^/NOS^+^/CRT^−^ (*single arrows*) and **l** FB^+^/NOS^−/^CRT^+^ (*small arrow*) neurons observed in Th3 neuromere. *Scale bar* image 1—120 μm; images 2, 4, 6, 11, and 12—100 μm; images 3 and 5—50 μm; image 7—25 μm; images 8, 9, and 10—20 μm

**Fig. 2:**
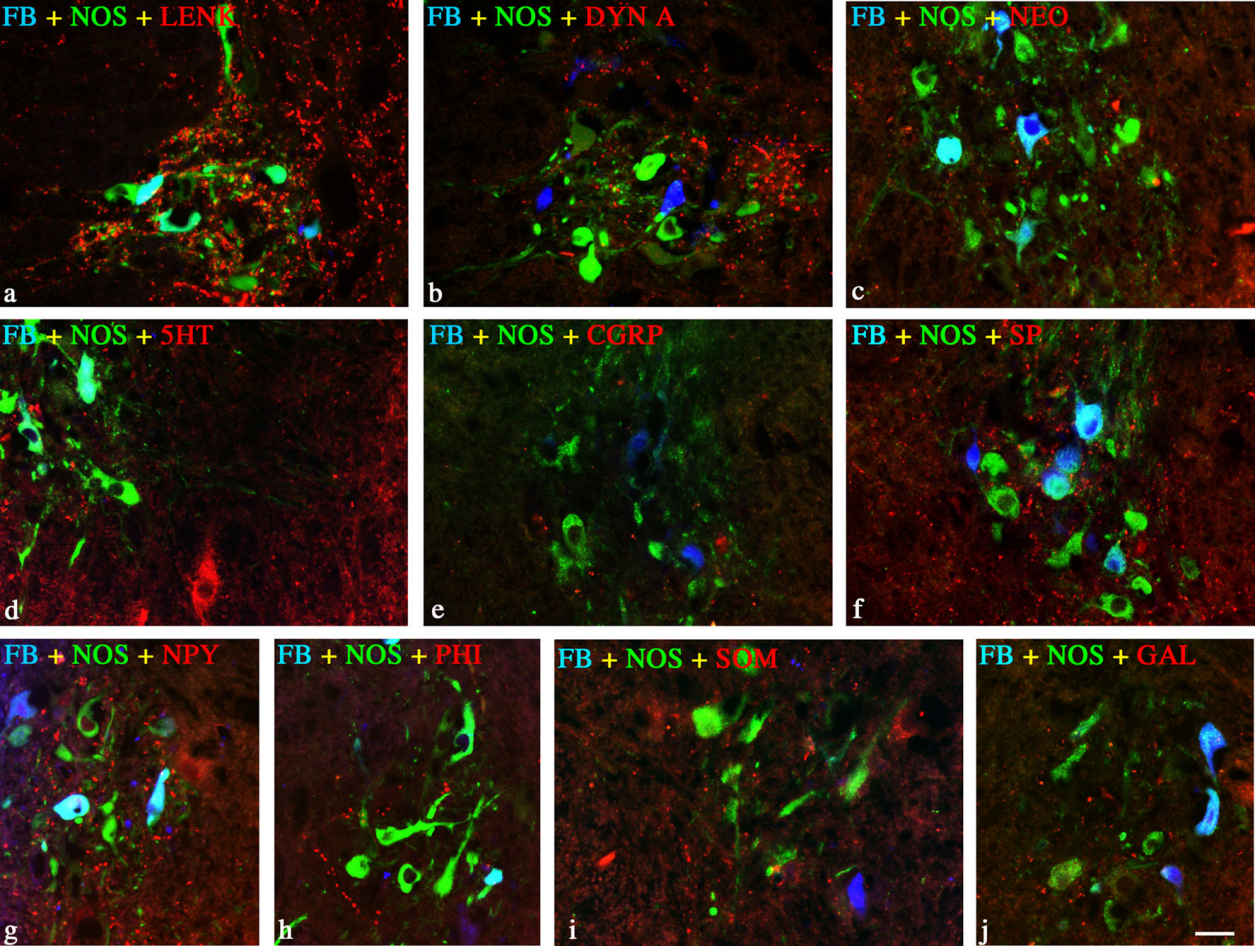
**a**—**j** Representative images of SCG-projecting FB+/NOS + neurons (SPN) and surrounding fibers observed in Th3 neuromere. All images are composites of merged images taken separately from the red, green, and blue fluorescent channels. Retrogradely labelled SPN were surrounded by **a** LENK-, **b** DYN A-, **c** α-NEO-IR, **d** 5HT-, **e** CGRP-, **f** SP-, **g** NPY-, **h** PHI-, **i** SOM-, **j** GAL-IR axons; *scale bar* 50 μm

FB neurons were localized predominantly in IMLpp (85.9 ± 2.4 %; 4,313 cells) and IMLpf (9.0 ± 1.5 %; 451 cells) and about 5 % of the cells were seen in ICpe (0.5 ± 0.2 %; 23 cells). ChAT-, NOS-, VAChT-, CRT-, CB-D28k-, SOM-, PACAP-, and CCK-immunoreactive FB^+^ neurons constituted approximately 88, 72, 7, 3.5, 3, 3, 1, and 0.6 % of all FB neurons in the whole region, respectively. The largest population of FB+/NOS + neurons was positive for ChAT (62.8 ± 4.9 %), followed by a much smaller number of VAChT (5.3 ± 2.7 %)-positive neurons. A few of the FB-positive nitrergic neurons also contained CB-28 k (2.2 ± 0.4), SOM (2.0 ± 0.7 %), and PACAP (1.0 ± 0.5 %) and a very limited number of FB+/NOS + neurons were positive to CCK (0.6 ± 0.4 %) and CRT (0.6 ± 0.4 %) (Table 2; Figs 1 and 2). None of the FB-positive perikarya was immunopositive to LENK, SP, PHI, NPY, VIP, GAL, CGRP, 5HT, and α-NEO. Retrogradely labelled SPN were surrounded by a very dense network of opioidergic (LENK-, DYN A-, or α-NEO-IR), GABA-, PACAP-, CART-, or VAChT-IR nerve terminals, a moderately dense network of SP-, SOM-, CALB-, CRT-, or TH-IR nerve fibers, and a scarce network of CCK-, VIP-, PHI-, NPY-, 5HT-, GAL-, or CGRP-IR axons (Figs. 1 and 2).

**Table 2:** Percentages of retrogradely labelled cells in sympathetic preganglionic neurons (SPN; in neuromers Th1–Th3) projecting to the porcine SCG

Substance	FB^+^/NOS^+^/P^+^	FB^+^/NOS^+^/P^−^	FB^+^/NOS^−^/P^+^	FB^+^/NOS^−^/P^−^
ChAT	62.8 ± 4.9	10.8 ± 1.2	16.2 ± 3.2	10.6 ± 1.4
VAChT	5.3 ± 2.7	65.9 ± 5.3	2.1 ± 1.1	26.1 ± 5.7
CB-28 k	2.2 ± 0.4	64.3 ± 4.5	0.8 ± 0.5	32.6 ± 4.9
SOM	2.0 ± 0.7	67.5 ± 1.3	0.8 ± 0.2	29.7 ± 1.5
PACAP	1.0 ± 0.5	66.0 ± 3.9	0.2 ± 0.2	32.7 ± 3.4
CCK	0.6 ± 0.4	67.1 ± 3.5	0	32.4 ± 3.6
CRT	0.6 ± 0.2	65.1 ± 5.7	2.9 ± 1.2	31.3 ± 5.3

P—substance (ChAT, VAChT, PACAP……..). Data expressed as mean ± standard deviation (SD)

## Discussion

Our results show that the majority of all neurons supplying SCG is localized between first through third spinal cord thoracic segments (TH_1–3_) and originates predominantly from nucleus intermediolateralis pars principalis and pars funicularis with only a small percentage found elsewhere, that is, in nucleus intercalatus spinalis pars paraependymali. These results are consistent with data obtained from other species (Dalsgaard and Elfvin 1979; Petras and Faden 1978; Yau et al. 1991) and provide further evidence establishing the predominant importance of intermediolateral nuclei in SCG innervation. The observation bears clinical relevance as intermediolateral column injury apparently disrupts SCG function, subsequently resulting in various vascular, respiratory, and cardiac dysfunctions (Grigorean et al. 2009; Krassioukov 2009; Popa et al. 2010). It has been shown that sympathetic IML morphological abnormalities in Th_1–3_ segments observed in patients with familiar dysautonomia lead to SCG shrinkage and activity suppression, resulting in numerous autonomic dysfunctions such as difficulties in swallowing, paresthesia, frequent vomiting, labile hypertension, propensity to lung infections, and corneal problems (Pearson and Pytel 1978); these findings further support the notion of central role of SCG in the autonomic coordination of major vital organs.

Further, our study revealed that the neurochemical composition of neurons supplying SCG is complex, underscoring the importance of this ganglion as a neuroendocrine center. First, we found that the majority of SCG preganglionic neurons is positive to both ChAT and NOS, showing a much higher level of colocalization than previously observed (Blottner and Baumgarten 1992; Calka et al. 2008).

ChAT, an acetylcholine marker, previously reported in the intermediolateral (IML) nuclei of several species (Całka et al. 2008; Ichikawa and Shimizu 1998; Markham and Vaughn 1990) is an important player in numerous neuromodulatory functions (Oda 1999). It has been found that disturbance in neuronal ChAT levels contributes to the mechanisms’ underlying development of certain neurodegenerative diseases such as Alzheimer’s or Huntington’s disorder, schizophrenia, or amyotrophic lateral sclerosis (Oda 1999). NOS, an indicator of nitric oxide presence in the neuron, was shown in the IML nuclei of a number of small and large laboratory animals as well as in humans (Calka et al. 2008; Foster and Phelps 2000; Lopez-Figueroa et al. 1996; Okamura et al. 1995; Reuss and Reuss 2001). It has been shown that NOS is involved in the regulation of several metabolic pathways such as soluble guanylyl cyclase or ADP-ribosyltransferase activation (Blottner and Baumgarten 1992) and, most importantly, modulates acetylcholine neurotransmission in central nervous system neurons affecting their activity (Prast and Philippu 2001). To date, only a few studies have indicated that in the IML column, NOS and ChAT co-exist in the same neurons (Burnett et al. 1995; Całka et al. 2008; Elfvin et al. 1997). It is plausible to assume that both NOS and ChAT play a vital role in the SCG preganglionic neurons, complementing each other’s functions and synergistically modulating neuronal activity of autonomic nervous system cells. Interestingly, VAChT, an acetylcholine transporter and another marker of cholinergic neurons, has been found in only 7 % of all SCG supplying neurons. This discrepancy between ChAT and VAChT distribution in cholinergic neurons might be explained by the differential genetic expression and diverse functional patterns of these two molecules at the molecular level (Weihe and Eiden 2000). ChAT and VAChT share the same genetic locus (Eiden 1998); however, it has been established that their expression levels might substantially differ and, depending on neuronal region and developmental stage, do not always correlate (Schutz et al. 2001), as observed in our study.

Furthermore, aside from ChAT, NOS, and VACHT, here for the first time we show the presence of a number of other neurochemicals such as CRT, CB-D28k, SOM, PACAP, and CCK in IML neurons innervating the SCG, indicating that these neurons largely contribute to and play an important role in maintaining the role of the SCG as a neuroendocrine center.

CRT has been identified in IML preganglionic sympathetic neurons of the rat (Murphy et al. 2003; Ren and Ruda 1994), mouse (Ninomiya et al. 1993), cat (Edwards et al. 1996), and lizard (gecko) (Morona et al. 2006). It belongs to the family of calcium-binding proteins and is involved in a wide array of processes from intracellular calcium signaling through molecular protein targeting to long-time potentiation modification (Schwaller 2014). It is also thought to be involved in neuroprotection, neuronal development, and homeostasis (Barinka and Druga 2010; Schwaller 2014). In the sympathetic nervous system, CRT is presumed to function as a regulator of sympathetic ganglion activity and as a potential contributor to age-related changes of target organ functions (Corns et al. 2000; Huerta et al. 1996).

CB, another member of the calcium-binding protein family, has been reported in the sympathetic preganglionic neurons of the rat’s IML column (Grkovic and Anderson 1997). In the sympathetic nervous system, CB has been speculated to contribute to the secretory functions of salivary glands and fatty brown tissues (Grkovic and Anderson 1997), play a role in the development of the enteric nervous system (Hagl et al. 2013), and contribute to the pathogenesis of proliferative enteropathy (Wojtkiewicz et al. 2012); however, its exact role has yet to be described. Recent studies indicate that CB may also play a role in excitatory activity of GABA and glutamate neurons projecting to IML sympathetic preganglionic neurons (Llewellyn-Smith et al. 2002), affecting their function.

CCK, first described as an intestinal hormone (Chandra and Liddle 2007), reported previously in rat, human, and guinea pig sympathetic neurons of IML nuclei (Chiba and Masuko 1987; Chung et al. 1989; Schroder 1983), has been shown to participate in a number of gastrointestinal processes, affecting the activity of numerous digestive system organs, i.e., stomach, gall bladder, pancreas, and intestines (Chandra and Liddle 2007). Studies have shown that changes in CCK expression or a reduction in a > number of CCK receptors contributes to various gastrointestinal and metabolic diseases such as diabetes mellitus, gall stone disease and irritable bowel syndrome (Chandra and Liddle 2007).

SOM, observed previously in the IML column of the guinea pig spinal cord (Chiba and Masuko 1989), has been shown to modulate inflammatory response between neuronal and mast cells in the intestine (Van Op den bosch et al. 2009) and likely acts alongside CCK in digestion and food intake (Schmidt et al. 1994; Zavros et al. 1998). It has been demonstrated that in the sympathetic nervous system SOM affects vasodepressor response (Rioux et al. 1981) and gastrin secretion (Olesen et al. 1984) and that it plays a role in insulin release (Lechin et al. 2013). It might be speculated that in the SCG, SOM plays a regulatory role, affecting the function of SCG target organs.

Finally, PACAP, previously found in SCG neurons of the IML column in the rat (Beaudet et al. 1998; Klimaschewski et al. 1996), is critically important in an array of autonomic nervous system-regulated activities, from metabolic and cardiovascular regulation through hormone secretion, stress response to appetite suppression, and food intake regulation (Merriam et al. 2013; Roy et al. 2013; Tanida et al. 2013). It has been shown that in the sympathetic nervous system, PACAP contributes to the neuronal development of the sympathetic neuroblast (Lu, et al., 1998) and modulates sympathetic neuron function via IP3, cyclic AMP/PKA, and MAPK/ERK regulatory pathways (Girard et al. 2004; May et al. 2000; Merriam et al. 2013). Furthermore, PACAP contributes to NPY and catecholamine expression in SCG neurons by regulating the mRNA levels of these neurotransmitters, thus affecting the function of this ganglion overall (Braas and May 1999; May and Braas 1995).

In addition to numerous neurochemicals present in SCG-projecting neurons, we found a dense network of neurochemically complex fibers surrounding the neurons and containing a plethora of neuropeptides such as LENK, DYN A, α-NEO, GABA, PACAP, CART, VAChT**,** SP, SOM, CALB, CRT, TH, CCK, VIP, PHI, NPY, 5HT, GAL, or CGRP that likely modulate the activity of SCG preganglionic neurons, thus regulating overall SCG function.

In conclusion, this is the first evidence of such a neurochemical complexity of IML preganglionic sympathetic neurons supplying mammalian SCG. It might be speculated that such complexity is indispensable to SCG activity and, as such, likely contributes to the central role of SCG in numerous crucial physiological and metabolic functions. The findings of our studies underscore the importance of SCG in the autonomic nervous system and lay the foundation for future translational and clinical studies.
